# Genotype- and Sex-Specific QT-RR Relationship in the Type-1 Long-QT Syndrome

**DOI:** 10.1161/JAHA.112.000570

**Published:** 2012-04-24

**Authors:** Jean-Philippe Couderc, Xiaojuan Xia, Isabelle Denjoy, Fabrice Extramiana, Pierre Maison-Blanche, Arthur J. Moss, Wojciech Zareba, Coeli M. Lopes

**Affiliations:** Center for Quantitative Electrocardiography and Cardiac Safety, Heart Research Follow-Up Program, University of Rochester Medical Center, NY (J.-P.C., X.X., A.J.M., W.Z.); Aab Cardiovascular Research Institute Department of Medicine, University of Rochester School of Medicine & Dentistry, NY (C.M.L.); INSERM-UMPC, Paris, France (I.D.); Hospital Bichat, Paris, France (I.D., F.E., P.M.-B.)

**Keywords:** electrocardiogram, KCNQ1, long-QT syndrome, QT interval, QT-RR dynamicity

## Abstract

**Background:**

Genotype-phenotype investigations have revealed significantly larger risk for cardiac events in patients with type 1 long-QT syndrome (LQT-1), particularly in adult females, with missense mutation in the cytoplasmic loop (C-loop) regions of the α subunit of the KCNQ1 gene associated with an impaired ion channel activation by adrenergic stimulus. We hypothesize that the impaired response to increases in heart rate leads to abnormal QT-RR dynamic profiles and is responsible for the increased cardiac risk for these patients.

**Methods and Results:**

We measured the QT-RR slope in 24-hour Holter ECGs from LQT-1 patients with the mutations associated with impaired adrenergic stimulus (C-loop, n=18) and compared to LQT-1 patients with other mutations (non–C-loop, n=48), and to a healthy control group (n=195). The diurnal QT-RR slope was less steep in C-loop mutation patients (0.10±0.05) than in the ECGs from non–C-loop mutation patients (0.17±0.09, *P*=0.002). For female patients, slower heart rates were associated with prolonged QT and increased QT-RR slope. Male patients with C-loop mutations showed an impaired repolarization for shorter range of heart rates than in females, which is consistent with gender differences in triggers for events in this syndrome.

**Conclusions:**

Our observations suggest that the C-loop LQT-1 patients have specific impaired adrenergic regulation of the ventricular repolarization. This response to heart rate increases may be useful in identification of high-risk patients with inherited prolonged QT and may help select an optimal antiarrhythmic therapeutic strategy. **(*J Am Heart Assoc*. 2012;1:e000570 doi: 10.1161/JAHA.112.000570.)**

## Introduction

The congenital long-QT syndrome (LQTS) type 1 (LQT-1) is associated with life-threatening arrhythmias and sudden cardiac death because of impaired cardiac repolarization due to mutations of the KCNQ1 gene encoding for the slow component of the repolarizing rectifier potassium currents (*I*_Kr_). LQT-1 represents one of the most clinically observed forms of the syndrome, accounting for 50% of all genotyped positive inherited LQTS.^1,2^ LQT-1 patients are at high risk for syncope during high adrenergic states such as exercise and emotion,^3^ and β-blockers are the treatment of choice for these patients. Efforts were made to better understand the underlying cause for life-threatening arrhythmias in LQT-1, ^4,5^ and a higher risk for cardiac events in LQT-1 patients carrying a mutation in the transmembrane region (S1 to S6) of the *I*_Kr_ channel were identified.^6^ In a more recent study, regions within the KCNQ1 potassium channel were formed based on cellular expression studies that revealed common mechanistic impairment and common clinical phenotypes. The work from Barsheshet et al^6^ suggested that the patients with missense mutations in the S2 to S3 and S4 to S5 cytoplasmic loop (C-loops) regions had a higher risk for events than patients with mutations outside of the C-loop regions. Furthermore, β-blockers were associated with greater benefit in patients with mutations located in the C-loop regions, supporting the role of an impaired β-adrenergic stimulation of the C-loop mutant channels as a factor contributing to an increased arrhythmic risk (A. Barsheshet, MD, submitted data, 2011). In addition, adult females with C-loop mutations seem to be at particular increased risk when compared to females with non–C-loop mutants (J. Costa, MD, submitted data, 2011).

The relationship between QT-interval duration and the immediately preceding RR interval, the so-called QT-RR dynamicity, has been shown to be influenced by various factors such as presence of drugs, heart rate, and more importantly the autonomic regulation of the heart.^7^ Sex effects on QT-RR dynamicity are known, with females showing a steeper QT-RR slope due to more prolonged QT at slow heart rates.^8^ An impaired β-adrenergic regulation of the ventricular repolarization is expected to modify QT-RR dynamicity. The modeling of the QT-RR relationship from continuous Holter recordings can be reliably computed,^9^ and the reproducibility of the modeling of the QT-RR dynamicity has been demonstrated.^10^ Previous investigations involving patients with the LQTS have revealed a steeper QT/RR slope than in controls, corresponding to an abnormal prolongation of the QT intervals at lower heart rate.^11,12^ However, cellular expression studies of mutant subunits of the KNCQ1 gene reported by Barsheshet et al (A. Barsheshet, MD, submitted data, 2011) revealed an association between C-loop mutations and impaired β-adrenergic regulation leading to a reduced adaptation of repolarization duration at high heart rate. In this work, we tested the hypothesis that LQT-1 patients with C-loop mutations present a different QT-RR dynamic profile than non–C-loop patients, which could explain variation of the benefit of β-blockers in LQT-1 patients and the different sex-associated arrhythmic triggers and risk for LQT-1 patients.

## Material and Methods

### Study Population

The Holter recordings from the genotyped LQTS patients were extracted from the Telemetric and Holter ECG Warehouse (THEW, http://www.thew-project.org),^13^ which is a repository for the sharing of fully deidentified ECGs. The THEW hosts multiple databases, including a set of four hundred and eighty 24-hour digital Holter recordings (Elatec Holter systems, ELA Medical) using 2- or 3-lead configuration recorded in 307 LQTS patients. This database was donated by the Hospital Lariboisière (Paris, France),^14^ and it includes genetic testing (including mutation), demographic data, and treatment (specifically β-blocker treatment at the time of the recording).^15^

In order to have a control group, we used the healthy database from the same repository that contains two hundred and two 24-hour Holter recordings from individuals without overt cardiovascular disease or history of cardiovascular disorders (including stroke, transient ischemic attack, and peripheral vascular disease), no history of high blood pressure (>150/90), no medication, and no other chronic illness (eg, diabetes, asthma, chronic obstructive pulmonary disease). The subjects were not enrolled if they were evaluated by a physician for cardiovascular-related syndrome (chest pain, palpitation, syncope). Their standard 12-lead ECG was without any suspicious abnormalities (eg, signs of ventricular hypertrophy, inverted T wave, intraventricular conduction disturbances). They all had normal echo and normal ECG exercise testing at the time of the ECG. No pregnant women were included. The Holter ECG recordings from the 2 groups of patients and healthy individuals are stored with a sampling frequency of 200 Hz and an amplitude resolution of 10 μV.

### Measurements

The ECG measurements were based on the COMPAS software developed at University of Rochester Medical Center (New York, USA). The software was applied to Holter ECG signals for all available leads, providing a beat-to-beat measurement of RR and QT intervals for the entire duration of each Holter ECG. QT-RR relationships were modeled by using a linear least-square fitting technique (MATLAB, Mathworks) for the diurnal (08:00 to 19:00) and the nocturnal (23:00 to 5:00) periods. The QT intervals were measured in the lead with the largest T-wave amplitude. QT intervals were determined to be unreadable when the T-wave amplitudes were <50 μV (flat T wave). The COMPAS software provided the location of the end of the T wave with a technique identifying the crossing point between the baseline and the slope fitting the terminal part of the T wave (least-squares technique). By default, the T wave with the highest amplitude across all leads was considered for measurements. The selection criteria did not consider any morphological features. The QT-interval measurements were computed in all available sinus beats by using an algorithm described previously.^16^ We visually reviewed the diurnal and nocturnal individual QT-RR scatterplots (including the linear fitting slope) to assess the quality of the measurements. Isolated and obviously wrong QT-RR couplets were filtered out before computing the linear fit. The corrected values of QT interval for heart rate (QTc) were computed by using the Bazett's formula. We computed the averaged QTc based on all single-beat measurements on the investigated period, that is, diurnal and nocturnal periods. Finally, we used time-domain parameters for estimating the role of the cardiac autonomic regulation as potential confounding factors in our multivariate models. SDNN (standard deviation of all normal-to-normal intervals) and RMSSD (square root of the means squared differences of successive RR intervals) measurements of heart rate variability were computed.

### Statistical Analysis

The statistical analysis was performed with the R statistical software suite. The tests for assessing the statistical differences in average values between investigated groups included the *t* test and nonparametric Cox-Wilcoxon tests. We used a multivariate linear regression model to investigate the influence of LQTS groups (C-loop:CL versus non-C-Loop:NCL mutations) and of potential confounding factors on the values of the QT-RR slope. These factors were age, sex, QTc, SDNN, and RMSSD parameters. The multivariate linear regression models were computed by using the LM functions from R. We investigated these models independently for 2 periods of the day: diurnal and nocturnal periods. The models were designed with an intercept, and estimated coefficients with a *P* value ≤0.05 were considered statistically significant.

## Results

### Study Population

We restricted our analysis to the Holter recordings from LQT-1 patients with missense mutation, who were off β-blockers and >13 years of age at the time of the Holter recordings. We identified 18 patients with mutation in the C-loops region (R174H/R174C, R190Q, R243H/R243C, V254M), 23 patients with mutation in C-terminus (A590T, I567T, R555C/R555H, and R591H), 1 patient with mutation in N-terminus (A46T, not reported), and finally 25 patients with non–C-loops membrane-spanning mutations (A344V, G168R, G269D/G269S, G314S, G325R, P320A, S225L, S277W/S277L, and Y315S/Y315C).

**Figure 1.**
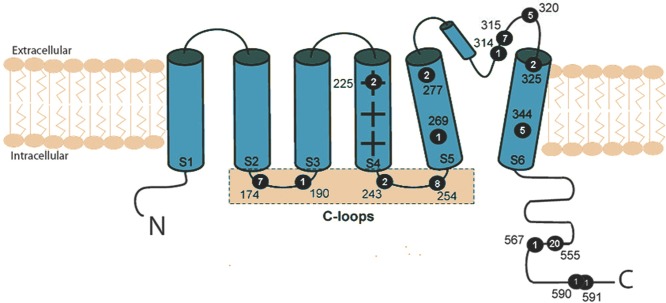
Distribution of the LQT-1 mutation locations within the KCNQ1 potassium channel of the study population. Schematic representation of the KCNQ1 potassium channel and the location of the mutations from the 66 patients with a recording off β-blockers investigated in this study. The number of patients per mutation is reported inside each black dot. Four mutations are located inside the C-loops (black dots between S2 to S3 and S4 to S5), 8 mutations are within the membrane-spanning segments, and 4 mutations in the C-terminus portion (C).

Figure 1 describes the location of these mutations within the KCNQ1 potassium channel and their numbers in our study population. After exclusion of the patients with mutation in the N-terminus, a total of 66 patients, 18 patients with mutations in the C-loop regions (CL LQT-1 group) and 48 patients with mutations in other locations (NCL LQT-1 group), were analyzed. One hundred and ninety-five Holter recordings from healthy individuals with age >13 years were identified. There was no difference in age between the 3 groups: controls, 39±15 years; CL LQT-1, 40±15 years; and NCL LQT-1, 39±16 years (*P*=0.5). The distribution of sex was not statistically different (*P*=0.81) between the groups: The controls had 49.7% women (97 females and 98 males), the CL LQT-1 group 61.1% women (11 females and 7 males), and the NCL LQT-1 group 60.4% women (29 females and 19 males). Thirty-three percent of CL patients and 37.5% of NCL patients had syncope (*P*=0.21). The remaining patients were all asymptomatic apart from a 36-year-old woman from the CL group who experienced a malaise.

We identified a second group of LQT-1 patients who were on β-blockers at the time of their Holter recordings. Twenty-six recordings, including 4 patients (3 women, 38±1 years) with a C-loop mutation and 25 patients (1 woman, 36±15 years) with a non–C-loop mutation, were analyzed. The numbers of patients per mutation in these groups were as follows: 1 G168R, 3 R174H/R174C, 2 S225L, 1 R243H, 2 S277W/S277L, 4 G314S, 2 Y315S/Y315C, 4 P320A, 3 G325R, 2 A344V, 4 R555C/R555H, and 1 R591H.

### Circadian ECG Changes in Controls and LQT-1 Patients off β-Blocker

We report the averages and the standard deviations of the QTc, RR, and QT-RR slopes measured in the recordings of the Control and LQT-1 patients (merging the CL and NCL groups, N=66) for the diurnal and nocturnal periods, respectively. The RR intervals were significantly shorter during the day than the night in Controls (741±100 versus 918±141 ms, *P*<0.0001), and in all LQT-1 patients (777±96 versus 947±144 ms, *P*<0.0001). The QTc was statistically different between diurnal and nocturnal periods in the control group (433±21 versus 427±26, *P*<0.001), confirming the observations about QTc in normal individuals,^9,10^ and in the LQT-1 patients (490±35 versus 483±27 ms, *P*=0.01). However, the QT-RR slope did not change between the 2 periods of the day (0.15±0.08 versus 0.15±0.08, *P*=0.9) in LQT-1, while in the control group this slope was steeper during the day than during the night (0.12±0.05 versus 0.10±0.05, *P*<0.0001). Therefore, we also confirm previous observations about the presence of an abnormal autonomic regulation of the ventricular repolarization in LQT-1 patients in comparison to controls. The LQT-1 patients have prolonged QTc interval and a more pronounced QT-RR slope than controls. This increased response of QT to RR interval was exacerbated during the nocturnal period.

### LQT-1, Mutation Region, and Gender in Patients off β-Blocker

As we show in Table 1, the average QTc-interval durations are not statistically different between the CL and NCL LQT-1 patients; the 2 groups exhibit rather pronounced QTc prolongation in comparison to Controls. However, the QT-RR slope is much steeper in NCL LQT-1 patients than in controls and CL LQT-1 patients. This steeper association between QT and RR values was significant for the diurnal period only.

**Table 1.** ECG Parameters for the CL and NCL LQT-1 Groups (Age >13 Years, Off β-Blocker)

	Controls (n=195)	CL LQT-1 (n=18)	NCL LQT-1 (N=48)	C vs CL	C vs NCL	CL vs NCL
Diurnal						

RR, ms	741±100*	802±131*	768±80*	0.07	0.03	0.7

QTc, ms	433±21*	495±28*	488±24*	<0.001	<0.001	0.3

QT/RR slope	0.119±0.052*	0.103±0.050	0.166±0.086	0.3	<0.001	0.002

Nocturnal						

RR, ms	918±141	930±157	954±140	0.7	0.07	0.3

QTc, ms	427±26	482±24	483±29	<0.001	<0.001	0.8

QT/RR slope	0.101±0.052	0.134±0.059	0.156±0.085	0.03	<0.001	0.3

* Significantly different values (*P*≤0.05) between diurnal to nocturnal values based on *t*-test and nonparametric Cox-Wilcoxon tests (Table 1).

**Figure 2.**
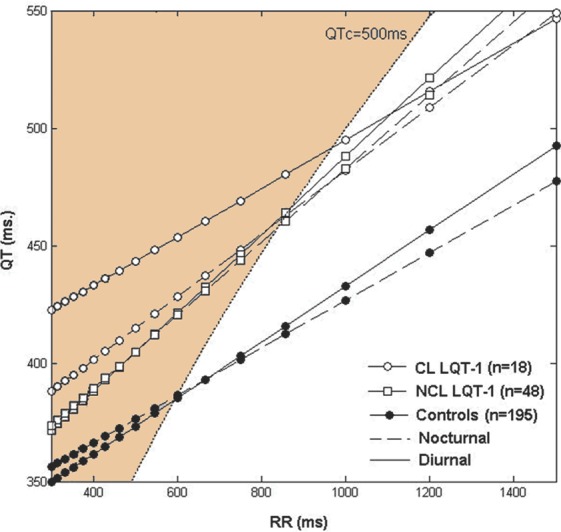
QT-RR profiles in the LQT-1 patients off β-blocker and in controls. QT-RR lines describing the QT-interval duration for the controls (black dots), CL QT-1 (white dots), and NCL LQT-1 (squares) groups for the diurnal (lines) and nocturnal (dotted lines) periods. We superimposed the curve describing QTc=500 ms, and grayed the areas corresponding to the range of RR and QT values associated with higher risk for cardiac events.

Interestingly, these CL LQT-1 patients exhibit a diurnal QT-RR slope equivalent to the control group (0.10±0.05 versus 0.12±0.05, *P*=0.3). In Figure 2, we illustrate the average QT-RR dynamicity profiles by plotting the QT relationship to RR values for heart rate varying from 40 to 200 bpm (beats per minutes) by step of 10 bpm. The figure highlights the profound changes in the dynamic profile of the QT interval between day and night periods in the CL LQT-1 patients, which is exacerbated at elevated heart rate. This observation is consistent with an impaired β-adrenergic regulation of *I*_Kr_ currents leading to a higher risk for arrhythmias.

The diurnal values of heart rate variability parameters did not evidence statistically significant differences between LQT-1 patients with mutation inside the C-loop region (SDNN: 101±36 ms and RMSSD: 34±10 ms) and the patients with mutations outside this region (SDNN: 109±42 ms and RMSSD: 37±23 ms). This observation was also valid for the diurnal period: The SDNN was 87±33 ms, and RMSSD was 39±17 ms for the CL group, while SDNN was 99±35 ms and RMSSD was 56±41 ms in the NCL group.

Using multivariate linear regressions, we investigated the association between QT-RR slope and the LQTS groups (CL and NCL groups) after adjusting for age, sex, QTc-interval duration, and heart rate variability estimators (see Table 2). We developed a set of models for both the diurnal and nocturnal periods. The model, including diurnal measurements of QT-RR slope as independent variable, revealed that NCL patients have 0.06 higher QT-RR slopes on average compared to CL groups (95% CI, 0.019–0.107; *P*=0.006) after adjusting for age, sex, QTc, SDNN, and RMSSD factors. Also, a significant association between the QT-RR slope and sex was found, corresponding to a 0.06 lower value in slopes if the patient was male for both groups NCL and CL (95% CI, 0.015–0.101; *P*=0.009). Interestingly, the model based on nocturnal values did not evidence such association between the QT-RR slope and the LQTS groups, which emphasizes the critical role of impaired β-adrenergic regulation of the repolarizing current(s) as the primary cause for QT prolongation.

**Table 2.** Description of the Multivariate Linear Regression Models Considering QT-RR Slope as Outcome When Comparing the Groups of LQT-1 Patients With Mutation Inside the C-Loop Regions (CL QT-1) or Outside These Regions (NCL QT-1)

	Diurnal Period			Nocturnal Period		
		
	Coefficient	95% CI	*P* value	Coefficient	95% CI	*P* value
Groups (NCL QT-1:1 vs CL QT-1:2)	0.063	0.019–0.108	0.0059	0.003	−0.014 to 0.007	0.194

Sex (male: 1 vs female: 2)	0.058	0.015–0.100	0.0093	0.002	−0.021 to 0.067	0.305

RR, ms	−0.0000	−0.0000 to 0.0000	0.974	−0.0002	−0.0004 to 0.0000	0.048

QTc, ms	0.0002	−0.0005 to 0.0011	0.530	0.0006	−0.0001 to 0.0013	0.136

RMSSD, ms	−0.0006	−0.0020 to 0.0007	0.385	−0.0004	−0.0012 to 0.0004	0.340

SDNN, ms	0.0006	0.0002–0.0014	0.137	0.0004	−0.0005 to 0.0001	0.416

Age, y	0.0010	−0.0005 to 0.0025	0.195	−0.0000	−0.00016 to 0.0001	0.898

Also, we applied logistic models considering the LQTS group as the outcome and diurnal QT-RR slope as an independent variable. The model revealed a significant association between the presence of mutation in C-loop region and a lower diurnal QT/RR slope (odds ratio: 1.16, 95% CI, 1.03–1.29; *P*=0.011) after adjusting for age, sex, QTc, SDNN, and RMSSD. A decrease of slope of 0.01 was associated with 16% increased likelihood for a patient to have a mutation in the C-loop region. Our objective is not to predict the location of the mutation based on ECG because we are studying genotyped LQT-1 patients specifically, but it is noteworthy.

**Figure 3.**
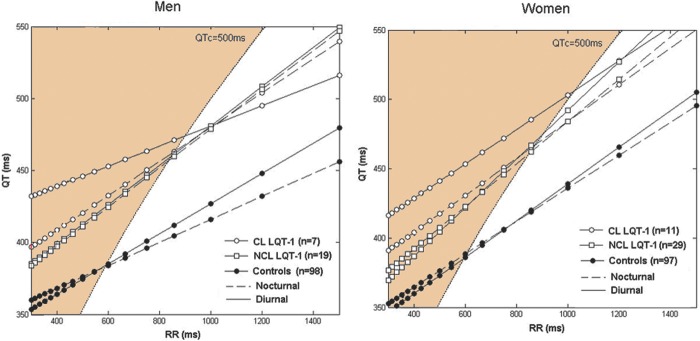
Sex- and mutation-location–specific QT-RR dynamic profiles in LQT-1 patients. We plotted the QT-RR profiles for men and women across all groups and circadian periods. Curves modeling the QT-interval duration for the controls (black dots), CL QT-1 (white dots), and NCL LQT-1 (squares) groups for the diurnal (lines) and nocturnal (dotted lines) periods are displayed. On average, the CL LQT-1 men have most abnormal QTc prolongation for elevated heart rate (above 70 bpm, RR≤857ms), while the range of heart rate with abnormal QT-interval duration is larger (above 60 bpm, RR≤1000 ms). We superimposed the curve describing QTc=500 ms, and grayed the areas corresponding to the range of RR and QT values associated with higher risk for cardiac events.

The comparison of the QT dependency to heart rate across sex confirms strong sex differences, as previously described by several authors.^9,17^ Our study provides new insights into differences of circadian changes between the sexes in healthy individuals. In healthy men, the QT-RR slope was significantly lower during the night than the day (0.08±0.04 versus 0.11±0.05, *P*=0.001), but this difference was weaker in healthy women (0.12±0.05 versus 0.13±0.05, *P*=0.02), which suggests a profound difference of the effect of male and female sexual hormones on the autonomic regulation of the ventricular repolarization. This is illustrated in Figure 3 (left panel). In addition, the diurnal QT-RR slope was significantly steeper in women than in men in the LQT-1 and the healthy groups. A larger QT prolongation at slower heart rates in women than in men explains this difference (see Table 3). For non–C-loop patients, this is partially compensated at higher heart rates by a strong decrease in QT at faster heart rates. For C-loop patients a concomitant decrease in shortening of QT at higher heart rates produces prolonged QT for female C-loop patients at a wide range of heart rates (above 60 bpm). The analysis of the recordings from patients on β-blockers does show very different profiles between CL (0.19±0.07) and NCL LQT-1 (0.17±0.04) groups (*P*=0.43). This group of patients on β-blockers was too small to assess the differences between genders.

**Table 3.** Sex-Specific QT-RR Dynamic Profile by Mutation Location in Controls and LQT-1 Patients

	Controls			CL LQT-1			NCL LQT-1		
			
	Male (n=98)	Female (n=97)	*P*	Male (n=7)	Female (n=11)	*P*	Male (n=19)	Female (n=29)	*P*
Diurnal									

RR, ms	753±93*	728±105*	0.08	842±162	759±100	0.24	765±80	743±79	0.19

QTc, ms	427±20*	439±21	<0.001	481±25	503±26	0.08	481±29	492±21	0.09

QT/RR slope	0.105±0.048*	0.132±0.053*	<0.001	0.070±0.055	0.124±0.037	0.05	0.137±0.075	0.175±0.080	0.05

Nocturnal									

RR, ms	941±142	895±137	0.02	1003±180	852±111*	0.08	911±137	965±142	0.30

QTc, ms	417±24	436±23	<0.001	480±24	484±23	0.21	479±30	484±25	0.08

QT/RR slope	0.08±0.045	0.119±0.054	<0.001	0.119±0.059	0.133±0.058	0.37	0.136±0.070	0.153±0.093	0.81

* Significance (*P*≤0.05) when compared to NCL LQT-1 group for same sex; *P*: *P* values when comparing means between genders.

## Discussion

We report an analysis of the QT-RR dynamicity in 24-hour Holter recordings acquired in healthy individuals and LQT-1 patients with and without mutation in the C-loop region. We observed stronger QT adaptation to heart rate in NCL LQT-1 patients regardless of the period of the day, whereas CL LQT-1 patients presented abnormal adaptation during the day driven by a very prolonged QT interval at elevated heart rate. Consequently, one expects CL LQT-1 patients to be at higher risk for cardiac events during elevated heart rate. This is consistent with the great benefit of β-blockers in this group^6^ and the type of arrhythmic triggers associated with high adrenergic stimulus.^18^

In the normal ventricular repolarization process, *I*_Kr_ is strongly activated by sympathetic stimulation and thought to be a major contributor to ventricular repolarization during adrenergic stimulation.^19^ LQT-1 mutations are associated with decreased *I*_Kr_ function, and consistent with the role of this channel during high sympathetic stimulation states, events in LQT-1 are mainly triggered during exercise and acute arousal.^20^ The proximal triggering mechanism in LQT-1 was described as tachycardia-induced torsades de pointes, a very different pathway than the pause-dependent trigger usually described in other LQTS mutations.^21^ During high adrenergic stimulation, heart rate is accelerated and cardiac contractility is increased, partially because of increases in calcium currents. Potassium currents, in particular *I*_Kr_, are important to shorten repolarization time and decrease the effective refractory period. C-loop mutations in *I*_Kr_ have been recently shown to be associated with a decrease in channel adrenergic activation,^22^ and this would be expected to result in an impaired ability of the channel to shorten QT intervals at fast heart rates. Recent studies showed a particularly increased risk for females with C-loop mutations (J. Costa, MD, submitted data, 2011), but in contrast, nonexercise triggers were associated with cardiac events in females in LQT-1.^23^ Our results can explain these disparate results, and suggest that a concomitant impairment of QT shortening at slow heart rates, possibly caused by inhibition of the *I*_Kr_ channel by sex hormones, combined with the decrease in response of patients to *I*_Kr_ adrenergic stimulus, is expected to cause an abnormally prolonged repolarization. Our results suggest that sex effects, possibly because of increased contribution of *I*_Kr_ to cardiac repolarization for females at lower heart rates, may have important implications for understanding sex-specific arrhythmic triggers in LQT-1.

A recent review by Goldenberg et al of data from 3386 genotyped subjects from 7 multinational LQTS registries revealed that genotype-confirmed patients with concealed LQTS (ie, normal QTc-interval duration) make up about 25% of the at-risk LQTS population. More importantly, this study showed that in mutation-positive subjects with normal-range QTc intervals, the genetic factors, including knowledge of the LQTS genotypes and the mutation location and type, identified patients who were at an increased risk for aborted cardiac arrest or sudden cardiac death (after adjustment for clinical variables).^24^ These clinical observations are consistent with the concept of plurality of the arrhythmogenic mechanisms involved in the triggering of life-threatening events in these LQTS patients and the limitation of the QTc-interval prolongation as a risk marker in these patients. The use of genotyping technologies represents the most accurate and reliable method to identify these patients. Still, genetic tests will never fully capture an individual risk that is modulated by exogenous factors. It is why scientists and clinicians have strived to develop techniques to improve the clinical identification and the risk stratification of LQTS patients. The use of quantitative electrocardiography remains vivid, and recent investigations proposed the response of the QT interval to brisk standing as a new diagnosis test,^25^ epinephrine challenge in LQT-1 patients,^26^ or abnormal T-wave morphology as complementary factor to QTc-interval prolongation in risk-stratifying cardiac events in LQT-2 patients.^27^ Here we showed sex-specific abnormal β-adrenergic regulation. Our study may be extended to identify patient-specific compensation to abnormal response of the ion channels. Impaired adaptation of the QT interval to RR may be relevant in the selection of optimal therapy for a patient before or after genotyping and identifying patient-specific arrhythmic triggers.

Precisely, our type of analysis could help identifying the nongenotyped LQT-1 patients at risk, but our results may also prove to be important in identifying patients with abnormal β-adrenergic regulation who may carry mutations outside of the C-loop region. These patients may carry an increase in risk and more effective response to β-blockers in a manner similar to the patients with C-loop mutations. Most importantly, within the group of C-loop patients, a steeper QT-RR may be associated with individual ability to maintain appropriate regulation because of other confounding genetic traits and may be associated with a lower cardiac risk. To demonstrate that such method has clinical value, one would need to investigate the association between life-threatening events and QT-RR slope values between the groups of patients with mutations inside and outside the C-loop regions. More generally one can speculate that other genetic or acquired ion channel characteristics that cause similar impaired QT-RR response may increase risk of drug-induced and heart-disease–associated arrhythmias.

## Limitation of the Study

If the QT-RR slope shows statistically significant differences between the groups of LQTS patients, one would emphasize that the slope measurements are associated with large interpatient variability within each group, reducing its interest as a diagnostic test. Furthermore, the data set used in this study is retrospective; thus the study was not powered for this specific analysis.

## Conclusions

LQT-1 patients with C-loop mutations have an abnormal regulation of ventricular repolarization consistent with an impaired β-adrenergic *I*_Kr_ regulation that is reflected by a less steep diurnal QT-RR slope measured from Holter ECGs.
